# Inverted U-shaped relationships between bone mineral density and VCTE-quantified degree of hepatic steatosis in adolescents: Evidence from the NHANES

**DOI:** 10.1371/journal.pone.0286688

**Published:** 2023-06-09

**Authors:** Shengmao He, Yun Zhang, Caixia Tan, Wenfu Tan, Bingliang Yin

**Affiliations:** 1 Department of Hand and Foot Surgery, The Affiliated Second Hospital, Hengyang Medical School, University of South China, Hengyang, China; 2 Department of Traumatic and Pediatric Orthopedics, The Affiliated Second Hospital, Hengyang Medical School, University of South China, Hengyang, China; Universitair Kinderziekenhuis Koningin Fabiola: Hopital Universitaire des Enfants Reine Fabiola, BELGIUM

## Abstract

**Introduction:**

There may be inaccuracies in hepatic steatosis in past research assessing the relationship between bone metabolism and liver steatosis. The goal of the current research was to look at the associations between bone mineral density (BMD) and the hepatic steatosis and fibrosis as detected by vibration-controlled transient elastography (VCTE) in teenagers in the United States.

**Methods:**

Weighted multiple linear regression models and smoothed curve fitting were used to investigate the association between BMD and the degree of hepatic steatosis and fibrosis in adolescents.

**Results:**

In 829 adolescents aged 12–19 years we found a negative association between total BMD and CAP (controlled attenuation parameter) [-32.46 (-58.98, -9.05)] and a significant positive association between lumbar BMD and LSM (liver stiffness measurement) [1.35 (0.19, 2.51)]. The inverted U-shaped relationships were founded between total BMD, lumbar BMD, pelvis BMD, and CAP with inflection points of 221.22 dB/m, 219.88 dB/m, and 216.02 dB/m, respectively.

**Conclusions:**

In adolescents, higher BMD is significantly associated with lower levels of hepatic steatosis and higher levels of liver stiffness.

## 1. Introduction

According to global research data, NAFLD has become one of the most common chronic liver diseases worldwide [1, 2], the prevalence of NAFLD has reached 25%, 24% and 23% in Asia, North America and Europe, respectively [3]. The prevalence of NAFLD is higher in people with metabolic syndromes such as obesity, diabetes and hyperlipidemia [4]. The continuation of liver steatosis in infancy into adulthood may be a cause of significant hepatic and metabolic illness, making early risk factor diagnosis and screening crucial [5].

Bone mineralization during adolescence is critical for preventing future osteoporosis and reducing fracture risk because during childhood or adolescence, the development and growth of bone mass is faster, so measures need to be taken to prevent osteoporosis as well [6, 7]. Although osteoporosis usually manifests itself in old age, a small percentage of people also develop it in childhood and adolescence [8]. Therefore, early prevention of osteoporosis in children and adolescents is of great significance to the growing public health challenge [9, 10]. Although research on the relationship between NAFLD and bone mineral density (BMD) in teenagers have been conducted [11, 12], these studies have almost always relied on index calculations to determine hepatic steatosis, an assessment method that is considered biased [13–15]. There is still a lack of strong evidence linking the bone metabolism with degree of hepatic steatosis and fibrosis in teenagers.

We therefore conducted a population-based study based on adolescents in the National Health and Nutrition Examination Survey (NHANES) to investigate a more accurate relationship between BMD with liver steatosis and fibrosis among US adolescents.

## 2. Methods

### Data source

**Fig 1:**
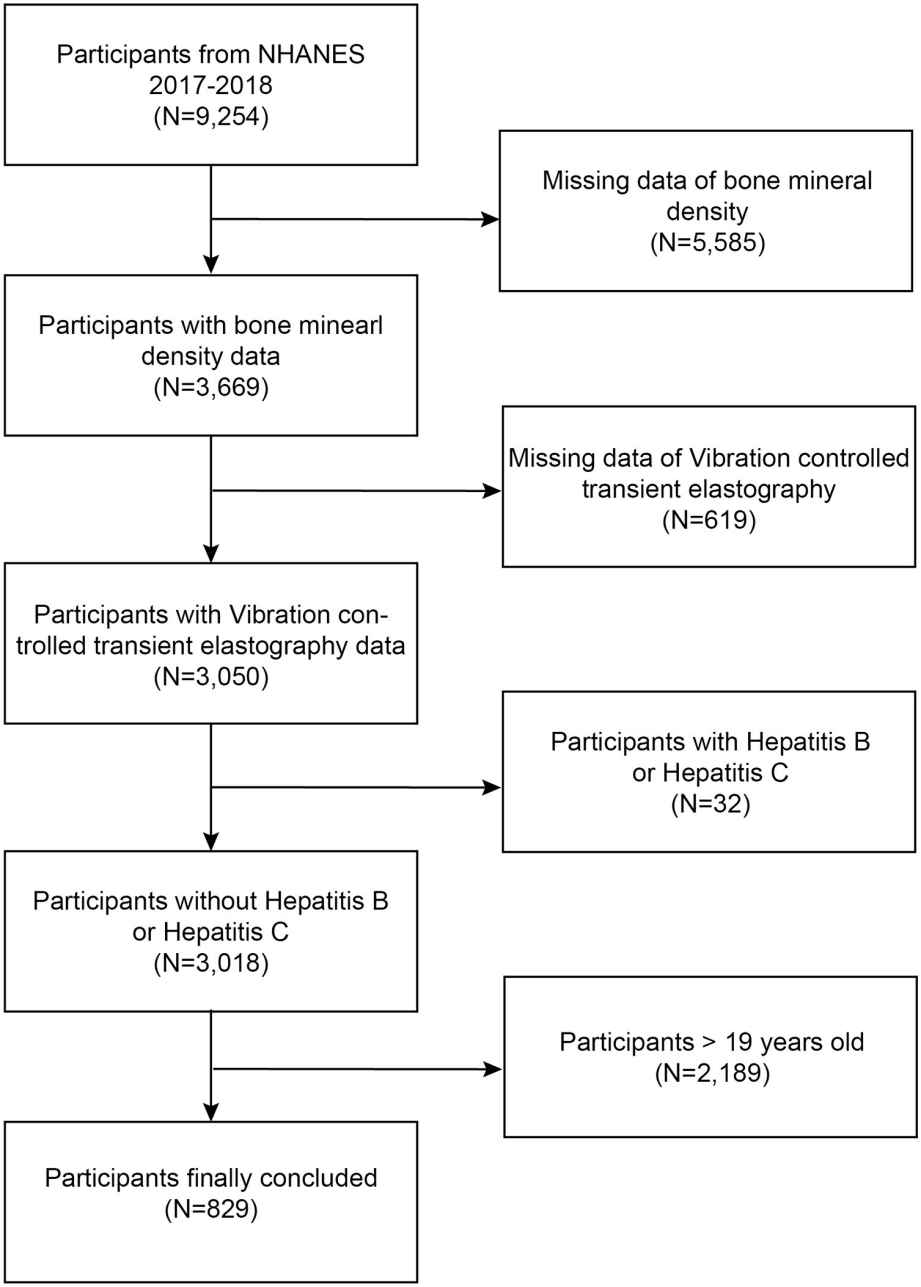
Flow chart of participants selection. NHANES, National Health and Nutrition Examination Survey.

NHANES is a large nationwide sample-based survey with a core component of investigating the nutrition and health status of adults and minors [16, 17]. The study procedure was approved by the National Center for Health Statistics (NCHS) Research Ethics Review Committee. The guardians of all minor individuals obtained written approval. Fig 1 illustrates the inclusion exclusions: 5585 participants without available BMD data, 619 participants with missing vibration control transient elastography (VCTE) data, 32 adolescents with viral hepatitis, and 2189 adults were excluded, resulting in the inclusion of 829 adolescent participants.

### VCTE

VCTE controlled attenuation data was used to determine the degree of hepatic steatosis and fibrosis. A skilled operator took at least ten times measurements from each subject, and the machine computed a median controlled attenuation parameter (CAP) [18]. The degree of fibrosis was determined by liver stiffness measurement (LSM) and optimized using the Jorden index [19].

### BMD

BMD in adolescents is measured by dual-energy X-ray (DEXA) scans, and in this study, we selected three sites for investigation (total BMD, lumbar BMD, and pelvic BMD).

### Covariates

Age, gender, race, ratio of family income to poverty, Vitamin D, high-density lipoprotein cholesterol, physical activities, BMI (body mass index), alkaline phosphatase, diabetes status, aspartate aminotransferase, high-sensitivity C-reactive protein, triglycerides, low-density lipoprotein cholesterol and total fat percent were all covariates in this study.

### Statistical analysis

R (version 4.2) and Empowerstats (version 5.0) were used for all analyses. The demographic parameters of the individuals by CAP quartile were assessed using the One-Way ANOVA test. The relationships between BMD and CAP and LSM were investigated using multivariate linear regression models. Smoothed curve fit (penalized spline approach) and two-segment linear models were used to explore the nonlinear association between BMD and CAP and LSM [20, 21]. To evaluate changes in the aforesaid relationships across gender, race, and age, subgroup analysis and interaction tests were utilized. Statistical significance was defined as a two-tailed P value of 0.05.

## 3. Results

### Baseline characteristics

At the time of assessment, the mean (SD) age of the 829 adolescents was 15.51 (2.26) years, with 52.747% of male participants. The weighted mean total BMD, CAP, and LSM were 1.04 (0.12) g/cm^2^, 221.76 (55.94) dB/m, and 5.01 (2.28) kPa, respectively. The characteristics of the study population according to the quartiles of the CAP are shown in Table 1. Adolescents in the higher quartiles of CAP were more likely to be Mexican Americans, to have a higher BMI and waist circumference, with higher pelvis BMD and LSM, and lower vitamin D levels.

**Table 1:** Basic characteristics of participants by CAP quartile.

Characteristics	Q1 (N = 205)	Q2 (N = 205)	Q3 (N = 211)	Q4 (N = 208)	*P*-value
Age (years)	15.44 ± 2.27	15.44 ± 2.34	15.68 ± 2.20	15.47 ± 2.25	0.656
Sex, n (%)					0.741
Male	103 (50.24%)	109 (53.17%)	108 (51.18%)	115 (55.29%)	
Female	102 (49.76%)	96 (46.83%)	103 (48.82%)	93 (44.71%)	
Race/ethnicity, n (%)					0.003
Non-Hispanic White	74 (36.10%)	82 (40.00%)	56 (26.54%)	49 (23.56%)	
Non-Hispanic Black	35 (17.07%)	40 (19.51%)	40 (18.96%)	36 (17.31%)	
Mexican American	35 (17.07%)	31 (15.12%)	41 (19.43%)	57 (27.40%)	
Other race/multiracial	61 (29.76%)	52 (25.37%)	74 (35.07%)	66 (31.73%)	
Diabetes, n (%)					0.429
Yes	1 (0.49%)	1 (0.49%)	4 (1.90%)	1 (0.48%)	
No	202 (98.54%)	203 (99.02%)	207 (98.10%)	205 (98.56%)	
Borderline	2 (0.98%)	1 (0.49%)	0 (0.00%)	2 (0.96%)	
Days of physical activities, n (%)					0.699
0–3	97 (47.32%)	81 (39.51%)	96 (45.50%)	92 (44.23%)	
4–7	108 (52.68%)	124 (40.49%)	115 (54.50%)	116 (55.77%)	
BMI (kg/m^2^)	21.03 ± 4.09	22.20 ± 3.71	23.97 ± 5.39	29.85 ± 6.89	<0.001
PIR	2.18 ± 1.51	2.13 ± 1.59	2.19 ± 1.57	1.97 ± 1.44	0.506
Triglycerides (mg./dL)	61.55 ± 32.63	68.31 ± 36.79	73.96 ± 58.81	89.38 ± 50.64	<0.001
HDL-C (mg/dL)	55.18 ± 10.99	55.09 ± 12.49	51.52 ± 10.78	47.94 ± 11.30	<0.001
LDL-C (mg/dL)	85.14 ± 27.05	85.20 ± 25.16	90.10 ± 22.70	94.62 ± 23.84	0.011
Waist circumference (cm)	74.48 ± 10.26	77.62 ± 9.51	81.28 ± 12.56	96.97 ± 16.73	<0.001
Vitamin D (nmol/L)	62.00 ± 24.22	60.47 ± 21.10	56.07 ± 21.46	51.23 ± 17.34	<0.001
Hs-CRP (mg/L)	2.08 ± 7.07	1.39 ± 3.60	1.92 ± 6.06	2.46 ± 3.56	<0.001
Total fat percent, (%)	26.63 ± 7.79	27.31 ± 8.37	29.81 ± 9.53	36.22 ± 7.81	<0.001
ALP (IU/L)	146.01 ± 103.44	151.48 ± 97.73	140.65 ± 96.03	149.10 ± 99.72	0.564
AST (U/L)	19.58 ± 5.96	20.14 ± 10.37	20.55 ± 14.32	21.79 ± 8.48	0.029
Blood urea nitrogen (mg/dL)	12.24 ± 3.41	11.93 ± 3.40	12.38 ± 3.96	11.51 ± 3.05	0.245
Pelvis BMD (g/cm^2^)	1.15 ± 0.18	1.17 ± 0.19	1.19 ± 0.18	1.25 ± 0.16	<0.001
Lumbar BMD (g/cm^2^)	0.97 ± 0.16	0.98 ± 0.16	0.98 ± 0.15	0.97 ± 0.14	0.667
Total BMD (g/cm^2^)	1.03 ± 0.12	1.04 ± 0.12	1.05 ± 0.12	1.05 ± 0.11	0.167
LSM (kPa)	4.61 ± 1.53	4.61 ± 1.21	4.73 ± 1.23	6.10 ± 3.73	<0.001

Mean ± SD for continuous variables: the P value was calculated by the weighted linear regression model.

(%) for categorical variables: the P value was calculated by the weighted chi-square test.

Abbreviation: Q, quartile; PIR, Ratio of family income to poverty; BMI, body mass index; HDL-C, High-density lipoprotein cholesterol; LDL-C, low-density lipoprotein cholesterol; CAP, Controlled Attenuation Parameter; LSM, Liver stiffness measurement; BMD, bone mineral density; Hs-CPR, high-sensitivity C-reactive protein; ALP, alkaline phosphatase; AST, aspartate aminotransferase.

### Association between BMD with hepatic steatosis and fibrosis

Table 2 presents the results of multivariate linear regression models between BMD with CAP and LSM. When not adjusted for any covariates, there are positive correlations between the three sites of BMD and CAP. However, in the partially adjusted model and the fully adjusted model, these associations shifted to negative correlations, particularly a negative association between total BMD and CAP, with each 32.46 dB/m decrease in CAP followed by a 1 g/cm^2^ increase in total BMD [-32.46 (-58.98, -9.05)]. The association between BMD and liver stiffness was positive in all models, with a positive association between lumbar BMD and LSM. The linear correlation between lumbar BMD and LSM remained significant after adjusting for all covariates, with each 0.84 kPa increase in LSM followed by a 1 g/cm^2^ increase in lumbar BMD [1.35 (0.19, 2.51)].

**Table 2:** The associations between bone mineral density with hepatic steatosis and fibrosis.

Bone mineral density	Model 1 [β (95% CI)]	Model 2 [β (95% CI)]	Model 3 [β (95% CI)]
**CAP (dB/m)**			
Total BMD (g/cm^2^)	48.19 (20.24, 75.61)	-34.01 (-62.30, -8.11)	-32.46 (-58.98, -9.05)
Lumbar BMD (g/cm^2^)	15.56 (-3.59, 36.76)	-30.54 (-58.69, -13.25)	-24.16 (-45.98, 1.36)
Pelvis BMD (g/cm^2^)	58.15 (31.97, 85.25)	-12.77 (-32.09, 8.83)	-6.30 (-25.71, 8.65)
**LSM (kPa)**			
Total BMD (g/cm^2^)	2.48 (1.25, 3.74)	1.27 (-0.09, 2.43)	1.27 (-0.37, 2.76)
Lumbar BMD (g/cm^2^)	1.77 (0.79, 2.75)	1.05 (0.03, 1.95)	1.35 (0.19, 2.51)
Pelvis BMD (g/cm^2^)	1.96 (1.12, 2.72)	0.74 (-0.05, 1.57)	0.85 (-0.19, 1.79)

Model 1: no covariates were adjusted. Model 2: age, gender, race, and BMI were adjusted. Model 3: age, gender, race, BMI, ALP, AST, PIR, Vitamin D, hs-CRP, total fat percent, physical activities, diabetes status, triglycerides, HDL-C and LDL-C were adjusted. Abbreviation: PIR, Ratio of family income to poverty; BMI, body mass index; HDL-C, High-density lipoprotein cholesterol; LDL-C, low-density lipoprotein cholesterol; CAP, Controlled Attenuation Parameter; LSM, Liver stiffness measurement; BMD, bone mineral density; Hs-CPR, high-sensitivity C-reactive protein; ALP, alkaline phosphatase; AST, aspartate aminotransferase.

In subgroup analyses stratified by gender, race, and age, the associations between BMD and LSM remained positive in all subgroups (Table 3). The associations between BMD and CAP were negative in all subgroups except race. In non-Hispanic blacks, the negative correlation changed into a positive, moreover, the results of the interaction test showed that race modified the association between BMD and CAP among adolescents (P for interaction < 0.05).

**Table 3:** Subgroup analysis of the association between total bone mineral density with hepatic steatosis and fibrosis.

Subgroup	CAP [β (95%CI)]	P for interaction	LSM [β (95%CI)]	P for interaction
Sex		0.098		0.361
Male	-21.75 (-51.18, 17.60)		1.20 (-0.41, 2.65)	
Female	-55.21 (-107.27, -2.92)		1.14 (-2.11, 4.38)	
Age		0.613		0.332
12–15	-24.11 (-65.33, 21.72)		2.09 (0.13, 4.01)	
16–19	-41.07 (-88.61, 2.60)		0.83 (-1.63, 3.51)	
Race/ethnicity		0.021		0.860
Non-Hispanic White	-3.71 (-59.22, 51.69)		1.69 (-1.71, 4.15)	
Non-Hispanic Black	30.65 (-49.93, 111.24)		1.31 (-2.68, 4.97)	
Mexican American	-140.28 (-222.80, -57.77)		0.76 (-3.13, 4.87)	
Other race/multiracial	-51.92 (-110.19, 5.20)		1.58 (-1.45, 5.11)	

Age, gender, race, BMI, ALP, AST, PIR, Vitamin D, hs-CRP, total fat percent, physical activities, diabetes status, triglycerides, HDL-C and LDL-C were adjusted. Abbreviation: PIR, Ratio of family income to poverty; BMI, body mass index; HDL-C, High-density lipoprotein cholesterol; LDL-C, low-density lipoprotein cholesterol; CAP, Controlled Attenuation Parameter; LSM, Liver stiffness measurement; BMD, bone mineral density; Hs-CPR, high-sensitivity C-reactive protein; ALP, alkaline phosphatase; AST, aspartate aminotransferase.

**Fig 2:**
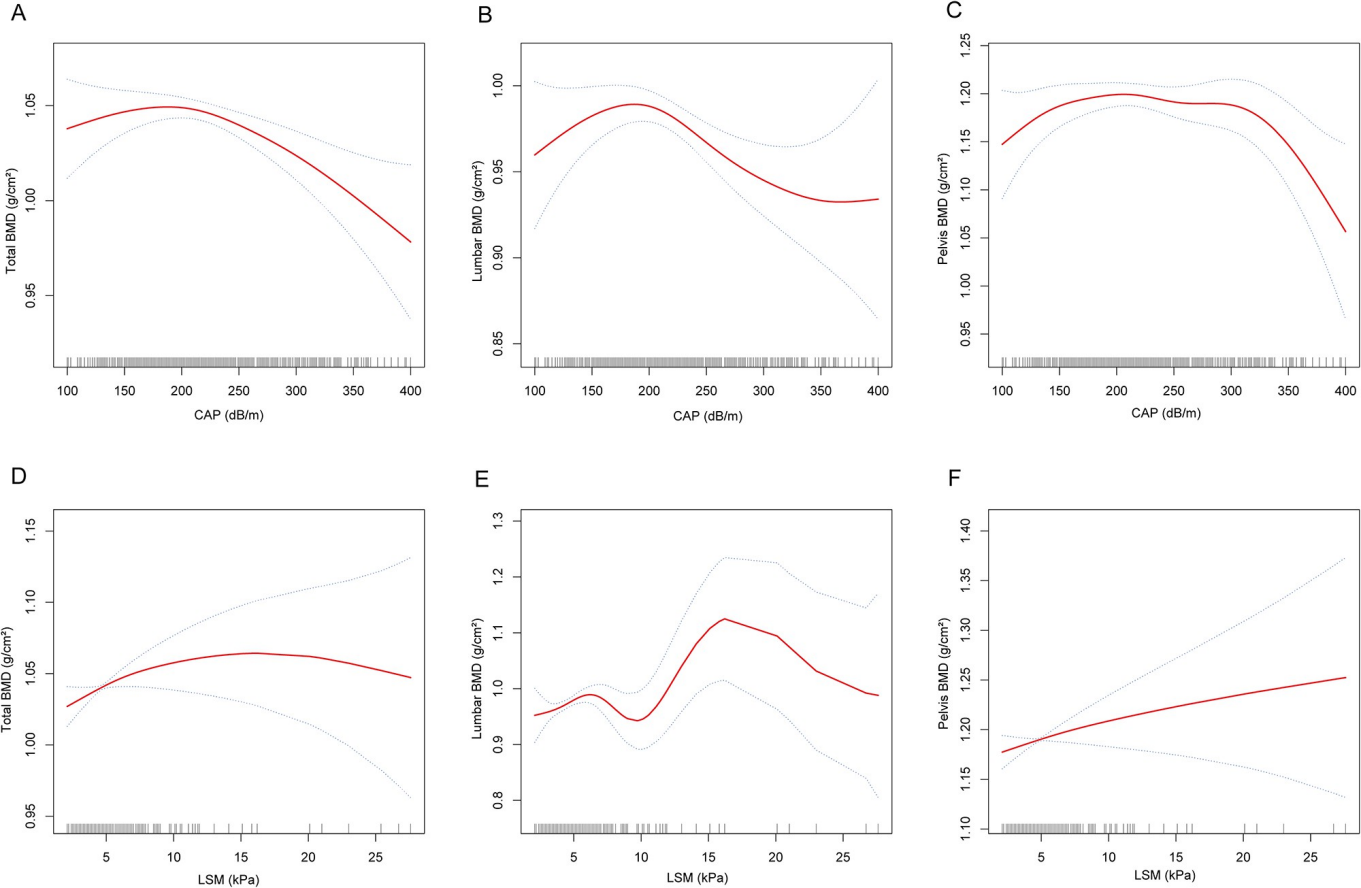
The nonlinear associations between BMD with degree of hepatic steatosis and fibrosis. The solid red line represents the smooth curve fit between variables. Blue bands represent the 95% of confidence interval from the fit. A. total BMD and CAP; B. lumbar BMD and CAP; C. pelvis BMD and CAP; D. total BMD and LSM; E. lumbar BMD and LSM; F. pelvis BMD and LSM.

In addition, Fig 2 illustrates the nonlinear relationship between BMD with CAP and LSM. The results indicated there are inverted U-shaped relationships between BMD and CAP at all three sites, and we subsequently used threshold effects analysis and two-segment linear regression models to further investigate the effects of inflection points and two segments in the inverted U-shaped relationship. The results showed that the inflection points of CAP for total BMD, lumbar BMD, and pelvis BMD were 221.22 dB/m, 219.88 dB/m, and 216.02 dB/m, respectively (Table 4).

**Table 4:** Threshold effect analysis of CAP on BMD by two-piecewise linear regression model.

CAP (dB/m)	Fitting by the standard linear model	Fitting by the two-piecewise linear model			
		Inflection point (K)	< K-segment effect	> K-segment effect	Log likelihood ratio
Total BMD (g/cm^2^)	-37.87 (-70.93, -4.81)	221.22	68.12 (-32.71, 168.95)	-61.88 (-101.30, -22.46)	0.029
Lumbar BMD (g/cm^2^)	-23.46 (-47.38, 0.45)	219.88	342.40 (118.91, 565.90)	-37.25 (-62.46, -12.04)	0.001
Pelvis BMD (g/cm^2^)	-5.16 (-26.30, 15.98)	216.02	45.09 (-11.99, 102.18)	-19.34 (-45.21, 6.53)	0.062

Models were adjusted for age, gender, race, BMI, ALP, AST, PIR, Vitamin D, hs-CRP, total fat percent, physical activities, diabetes status, triglycerides, HDL-C and LDL-C were adjusted. Abbreviation: PIR, Ratio of family income to poverty; BMI, body mass index; HDL-C, High-density lipoprotein cholesterol; LDL-C, low-density lipoprotein cholesterol; CAP, Controlled Attenuation Parameter; LSM, Liver stiffness measurement; BMD, bone mineral density; Hs-CPR, high-sensitivity C-reactive protein; ALP, alkaline phosphatase; AST, aspartate aminotransferase.

## 4. Discussion

In this study, we assessed the associations between BMD with the degree of hepatic steatosis and fibrosis in U.S. adolescents and found that lower CAP and higher LSM were significantly associated with higher BMD. In addition, we identified an inverted U-shaped correlation between BMD and CAP and the point of infection, which may further explain the potentially complex association between the degree of hepatic steatosis and fibrosis and bone metabolism in adolescents. To our knowledge, this is the first study that investigated BMD in adolescents in relation to the hepatic steatosis and liver stiffness quantified by VCTE.

Past epidemiological studies investigating BMD and NAFLD in adolescents have been few and inconsistent. A study from Turkey included 82 adolescents with obesity and 30 control participants and showed that NAFLD has a detrimental effect on bone health in adolescents and is associated with increased insulin resistance [22]. However, in this study, as most epidemiological studies investigating NAFLD have used indices or ultrasound to diagnose the severity of hepatic steatosis, emerging evidence suggests that the results of indices assessment underestimate the prevalence of NAFLD [23, 24]. A META analysis that included 8 epidemiological studies of 632 children and adolescents who underwent VCTE or biopsy investigated the relationship between NAFLD and decreased BMD in children, and the results of a random effects model suggested that the presence and severity of NAFLD were significantly associated with reduced whole-body BMD Z scores in children and adolescents [25]. In a recent cross-sectional study, also based on U.S. adolescents, investigating the association between BMD and NAFLD diagnosed by liver enzyme calculations, the authors concluded that adolescents with NAFLD had higher BMD and found gender and racial differences in this association [11]. Our findings also suggest significant differences between non-Hispanic blacks and participants of other races in the association between BMD and hepatic steatosis. In fact, gender and ethnic differences are common in epidemiological studies on BMD [26]. For example, in the association between lipid metabolism and BMD, Xie et al. found a significant nonlinear association between HDL-C and BMD in men and whites, but not in blacks [27], and a negative linear association between LDL-C and lumbar BMD only in whites and Mexican Americans, but not in blacks [28]. However, after reviewing the past literature, we found that the potential mechanism regarding this situation is currently unexplained. In addition, the association of BMD with CAP and LSM differed across age subgroups, which likely stems from dynamic changes in bone and liver metabolism during adolescent growth and development [29].

According to the current study, there are several hypotheses for a significant association between BMD and the degree of hepatic steatosis and fibrosis. First, some investigators have suggested that insulin resistance is the main mediator of this relationship and that insulin resistance affects bone metabolism by influencing the increase or decrease of multiple inflammatory metabolic factors and bone metabolites, which leads to an acceleration of bone density loss [30, 31]. Pacifico et al. demonstrated in a case-control study that CRP and other inflammatory mediators may accelerate bone mass loss in adolescents with NAFLD [32]. In addition, past studies on TNF-α have demonstrated its ability to inhibit osteoblast differentiation and enhance osteoclast activity and osteoblast apoptosis [33–35]. More importantly, the relationship between BMD and the degree of hepatic steatosis and fibrosis is largely mediated by obesity and body fat content, and adolescents with obesity may help prevent bone loss by increasing mechanical load [36, 37]. This was also verified in our multivariate linear model, where there was even a positive association between BMD and CAP when unadjusted for BMI.

There are various limitations to our research. First, due to the limitations of the cross-sectional investigation, we were unable to show a causal relationship between BMD and the degree of hepatic steatosis and fibrosis. Furthermore, because we excluded teenage individuals with viral hepatitis, our findings in this cohort should be regarded with care. Despite these flaws, our research offers some advantages. In this study, we used VCTE findings to more accurately quantify the degree of hepatic steatosis and fibrosis in teenagers than index estimates. More importantly, we discovered a substantial inverted U-shaped link between BMD and the degree of hepatic steatosis in adolescents, as well as an inflection point that may assist clinicians and public health officials avoid osteoporosis in adolescents.

## 5. Conclusion

According to our findings, BMD is negatively connected to the degree of hepatic steatosis and positively related to LSM in teenagers.

## Abbreviations

BMI: body mass index
CAP: controlled attenuation parameter
DEXA: dual-energy X-ray
LSM: liver stiffness measurement
NAFLD: non-alcoholic fatty liver disease
NCHS: National Center for Health Statistics
NHANES: National Health and Nutrition Examination Survey
VCTE: vibration-controlled transient elastography
